# Estimated lifetime risk of venous thromboembolism in men and women in a Danish nationwide cohort: impact of competing risk of death

**DOI:** 10.1007/s10654-021-00813-w

**Published:** 2021-11-08

**Authors:** Carl Arne Løchen Arnesen, Katalin Veres, Erzsébet Horváth-Puhó, John-Bjarne Hansen, Henrik Toft Sørensen, Sigrid K. Brækkan

**Affiliations:** 1 Thrombosis Research Center (TREC), Department of Clinical Medicine, UiT-The Arctic University of Norway, 9037 Tromsø, Norway; 2 Department of Clinical Epidemiology, Aarhus University and Aarhus University Hospital, Aarhus, Denmark; 3 Division of Internal Medicine, University Hospital of North Norway, Tromsø, Norway

**Keywords:** Epidemiology, Estimated life time risk, VTE, DVT, PE, Competing risk of death, Cumulative incidence, Reproductive risk factors

## Abstract

Incidence of venous thromboembolism (VTE) risk varies by age and sex. Some studies have reported overall higher risk in men, especially when VTEs triggered by female reproductive factors are excluded. However, higher mortality rates in men may have led to overestimation of lifetime VTE risk in men compared with women. Therefore, we estimated the lifetime risk of VTE in men and women in a Danish, nationwide cohort, taking into account the competing risk of death. Within the population of Denmark (> 5 million persons), all first-time VTEs occurring in 1995–2016 were identified from the Danish National Patient Registry covering all Danish hospitals. The cumulative incidences of VTE were estimated in men and women with age as timescale, taking into account the competing risk of death. Estimated lifetime risk was defined as cumulative incidence at age 100. In a simulation study, we excluded the proportion of female cases that could be attributed to reproductive risk factors and re-estimated the cumulative incidence. We identified 123,543 incident VTEs. The cumulative incidence of VTE was 1.9% in women and 1.3% in men at age 50, 4.3% in women and 4.4% in men at age 70, and 9.3% in women and 8.1% in men at age 100. After accounting for VTEs attributed to reproductive factors, the corresponding incidences in women were 1.2% at age 50, 3.2% at age 70, and 8.2% at age 100. In conclusion, the estimated lifetime risk of VTE was slightly higher in women than in men when accounting for competing risk of death. Our simulation study suggested that reproductive risk factors contribute modestly to the estimated lifetime VTE risk in women.

**Supplementary Information:**

The online version contains supplementary material available at 10.1007/s10654-021-00813-w.

## Introduction

Previous studies of venous thromboembolism (VTE) risk by sex have provided inconsistent results, with some studies reporting a higher risk in women [1], some reporting a higher risk in men [2–4], and others finding no difference [5]. Incidence rates of VTE in men and women are also reported to vary with age, and differences in study results for overall VTE in men versus women are likely explained by differences in the age range of the populations under study [2, 3, 6, 7]. In younger age groups (< 50 years) the incidence of VTE is higher in women than in men [7] due to female reproductive risk factors (e.g. oral contraceptives and pregnancy), while in middle-aged persons (50–70 years) the incidence is higher in men than in women [2, 6].

A case–control study of persons aged 18–70 years reported that when female reproductive factors were taken into account, the odds ratio was twofold higher in men than in women [8]. This has led to the hypothesis that men have a higher intrinsic risk of incident VTE than women [8]. However, female reproductive risk factors are mainly present at young age (< 50 years), when the baseline risk of VTE is low, thus resulting in a large relative, but modest absolute risk increase. To date, no study has assessed VTE risk in men and women over the lifespan, accounting for the contribution of female reproductive risk factors to lifetime risk in women.

The competing risk of death is another factor that can influence VTE risk differences in men and women [9]. Men have a higher mortality rate than women across all age groups [10, 11]. This may result in overestimation of VTE risk in men compared with women, particularly in the older age groups, which have the highest absolute difference in mortality rates by sex [12]. Thus, the apparently higher VTE risk in middle-aged and older men could potentially be explained by differences in mortality rates. We are aware of only one study that assessed the lifetime risk of VTE in men and women, while accounting for the competing risk of death. Using two US cohorts aged 45–85 years, Bell et al. found marginally higher cumulative VTE incidence in women than in men (8.4% vs. 7.7%) [13]. To our knowledge, no study has assessed the age-specific cumulative incidence of VTE in men and women using a nationwide cohort including subjects of all ages and taking into account the competing risk of death.

The aim of this study was to estimate the lifetime risk of VTE, deep vein thrombosis (DVT), and PE among men and women using a Danish nationwide cohort, and accounting for death as a competing event. Furthermore, to examine the hypothesis that the inherent lifetime risk of VTE is higher in men, we simulated how absence of female reproductive risk factors could influence the lifetime risk of VTE in women compared with the lifetime risk in men.

## Methods

### Setting and study population

Our study was based on data obtained from Danish healthcare and administrative registries. The Danish healthcare system is government-funded and provides all legal residents with free access to health care [14]. Complete individual-level linkage between all health and administrative registries is enabled by the unique 10-digit identification number assigned by the Danish Civil Registration System (CRS) to all legal residents at birth or upon immigration [15]. The Danish National Patient Registry (DNPR), which is linked to the CRS, contains data on more than 99% of all inpatient stays and outpatient visits to Danish hospitals. Each hospitalization or outpatient visit is recorded in the DNPR with one primary diagnosis and one or more secondary diagnoses coded according the *International Classification of Diseases, Tenth Revision* (ICD-10) [16].

The entire population of Denmark in 1995 (*N* = 5,306,416) served as the source cohort. Individuals with a history of VTE before 1995 were excluded. The cohort was followed from January 1, 1995 through December 31, 2016. Information on age, sex, date of emigration (when applicable), and death (when applicable) was obtained from the CRS.

### Ascertainment of patients with venous thromboembolism

The DNPR was used to identify all inpatients and outpatients with a primary or secondary discharge diagnosis of DVT, PE, splanchnic vein thrombosis, or cerebral vein thrombosis, based on ICD-10 diagnosis codes (listed in Supplementary Table 1), during the period January 1, 1995 through December 31, 2016. In patients experiencing DVT and PE simultaneously, only PE was counted due its greater severity.

### Competing risk of death

Non-informative (i.e. independent) censoring is an assumption in the traditional Kaplan–Meier survival analysis. This means that at each time point, subjects who remain in the study should have the same future risk of the disease (i.e. VTE) as those who have dropped out of the study (censored). However, when a person is censored due to death, the risk of VTE is instantly reduced to zero, and the assumption of non-informative censoring is therefore violated. Consequently, a high risk of death will lead to an overestimation of VTE risk, and thus bias the results [17, 18]. The cumulative incidence function (CIF) allows for estimation of the incidence of an outcome while taking competing risk into account. In the competing risk setting, only one type of event can occur. Thus, the occurrence of one event precludes subsequent occurrence of other event types. The sum of the CIF estimates for each separate outcome equals the CIF estimate for the composite outcome comprising all the competing events [17].

### Statistical analysis

We characterized the source cohort according to sex and age groups. Person-time of follow-up accrued from inclusion in the cohort until the date of an incident VTE, emigration from Denmark, death, or end of the study period (Dec 31, 2016), whichever occurred first. To estimate incidence rates of VTE and death, the population was divided into five-year age groups ranging from 0–4 to 100 + years. Age was used as the time scale, and the 5-year age groups were modeled as time-varying covariates (*i.e*., individuals contributed person-time to different age groups as they grew older during the study period). Incidence rates (IRs) with 95% confidence intervals (CIs) for overall VTE, DVT, PE and death were calculated for each 5-year age group by dividing the number of VTEs by the person-time accrued in that specific age group in men and women separately. These IRs were expressed as number of events per 1000 person-years (PY) at risk.

Cumulative incidence curves with and without death as a competing event were estimated using age as the time scale. Cumulative incidence with death as a censoring event was estimated using the complement of the Kaplan–Meier product limit estimator (1-KM), while the cumulative incidence estimate with death as a competing event was calculated using CIF [19]. The estimated lifetime risk was defined as the CIF-estimate at age 100.

Since the incidence of VTE has increased during the last 20 years [20], we performed sensitivity analyses to investigate potential birth cohort effects in men and women. The study period was divided into 1995–2005 and 2006–2016, and cumulative incidence curves for men and women were created for each interval.

### Simulation study to account for female reproductive risk factors

During their lifetimes, most women are exposed to risk factors associated with female reproduction, such as hormonal contraception or pregnancy, which is known to increase the risk of VTE [21]. We therefore wished to simulate how absence of female reproductive risk factors would influence the cumulative incidence of VTE in women. Studies using relative measures have reported a twofold higher risk of VTE in men compared to women without reproductive risk factors [7, 8]. In a nationwide study of Danish women aged 15–49, Lidegaard et al. reported an overall IR of VTE of 4.03 per 10,000 woman-years in the total population [22]. The IR among former or never users of oral contraceptives was 3.01 per 10,000 woman-years, while the IR among current oral contraceptive users was 6.29 per 10,000 woman-years [22]. Based on these rates, the population-attributable fraction (PAF) of oral contraceptives would be 25% in the 15–49-year age group. Similarly, a nationwide Danish study of pregnancy-related VTE in women aged 15–49 reported that the IR among non-pregnant women was 3.6 per 10,000 woman-years, while the IR among pregnant women was 10.7 per 10,000 woman-years [23]. This corresponded to an estimated PAF of pregnancy of 10%. We estimated conservatively that 45% of all VTE cases in the 15–49 age group could be attributed to oral contraceptive use or pregnancy. In our simulation, we therefore randomly treated 45% of the VTE cases in this age group as if they had never occurred (*i.e*., the proportion of female cases that could be attributed to reproductive risk factors) and re-estimated the CIF of VTE in women. The simulation approach was repeated 10 times, to obtain an average cumulative incidence measure from the 10 simulations.

SAS version 9.4 (SAS Institute Inc. Cary, North Carolina, USA) was used to perform the statistical analyses and the simulation.

## Results

**Fig. 1:**
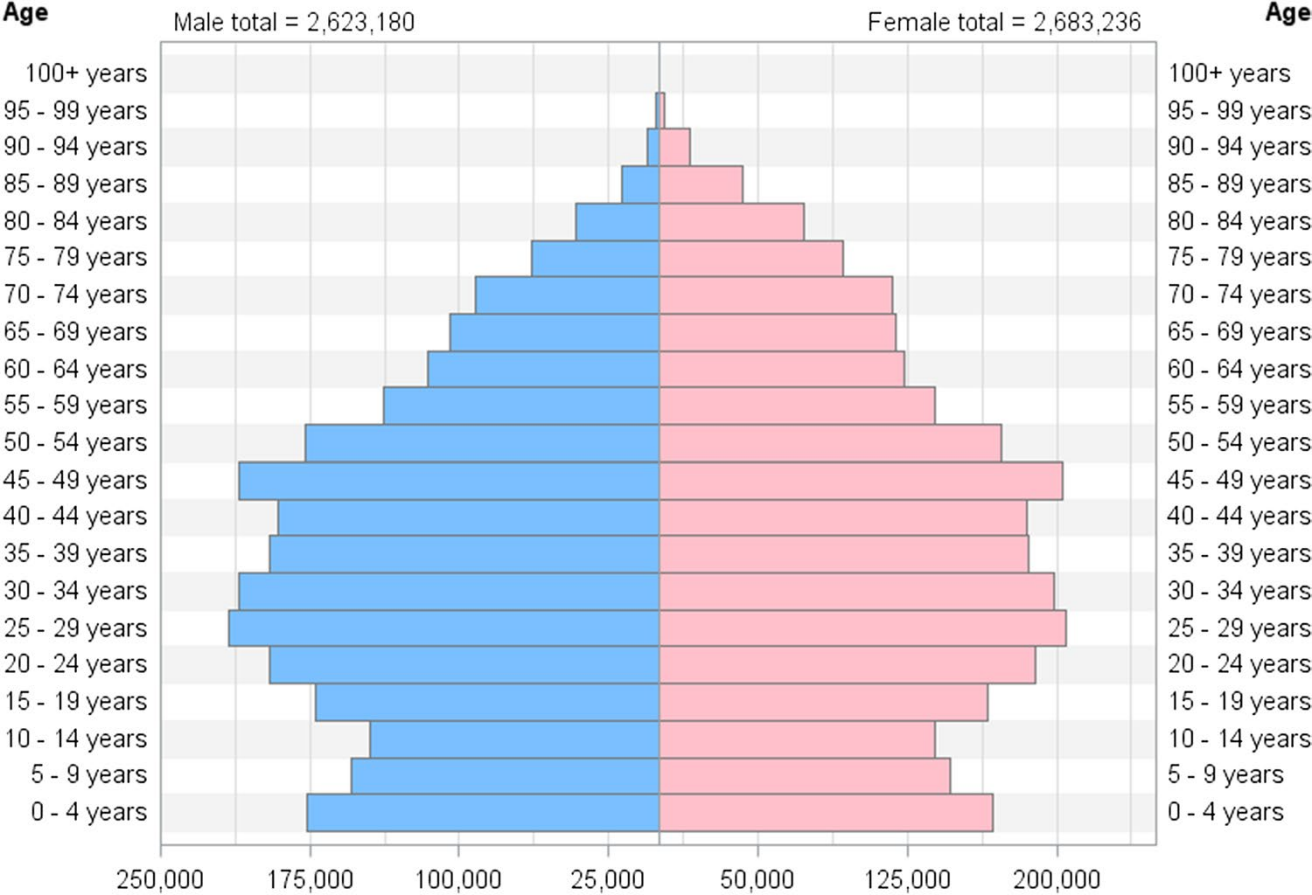
Distribution of the study population at baseline inclusion in 1995 by sex and five-year age groups

Our study cohort consisted of 2,623,180 (49.4%) men and 2,683,236 (50.6%) women. We identified 123,543 individuals with a first-time diagnosis of VTE between January 1, 1995 and December 31, 2016. The baseline distribution of the study population by 5-year age groups in men and women is presented in Fig. 1. Among the VTEs, 73,749 (59.7%) were DVTs and 49,794 (40.3%) were PEs. The proportion of VTEs presenting as PEs was 41.4% in women and 39.1% in men.

### Incidence rates of VTE

The overall crude incidence rates of VTE were 1.28 (95% CI 1.27–1.29) per 1000 person-years in women and 1.17 (95% CI 1.16–1.18) per 1000 PY in men. The incidence rates increased with age in both men and women. In the 15–45-year age group, the incidence rates were higher in women than in men, ranging from 0.24 to 0.68 per 1000 PY in women and from 0.07 to 0.62 per 1000 PY in men (Table 1). For persons aged 45 to 80 years, the rates were higher in men than in women (ranges: 0.85–4.16 per 1000 PY in men and 0.79–3.94 per 1000 PY in women). In the oldest age groups (≥ 80 years) the rates were similar or slightly higher in women than in men, ranging from 4.94 to 5.73 per 1000 PY in women and from 4.92 to 4.98 per 1000 PY in men (Table 1). The overall incidence rate of DVT was 0.75 (95% CI 0.74–0.76) per 1000 PY in women and 0.71 (95% CI 0.70–0.72) per 1000 PY in men. The rate of PE was 0.53 (95% CI 0.52–0.54) per 1000 PY in women and 0.46 (95% CI 0.45–0.47) per 1000 PY in men. The incidence rates of DVT and PE followed the same patterns as those observed for overall VTE in men and women across all age groups (Supplementary Tables 1 & 2).

**Table 1:** Incidence rates per 1000 person-years of venous thromboembolism by five-year age groups in men and women

	Women			Men		
Age (years)	n	Events	IR (95% CI)	n	Events	IR (95% CI)
0–4	167,754	2	0.00 (0.00–0.01)	176,096	0	–
5–9	313,930	5	0.00 (0.00–0.01)	329,681	7	0.01 (0.00–0.01)
10–14	451,067	25	0.01 (0.01–0.02)	472,786	17	0.01 (0.00–0.01)
15–19	614,941	620	0.24 (0.22–0.25)	643,523	194	0.07 (0.06–0.08)
20–24	799,829	1355	0.40 (0.38–0.43)	835,050	461	0.13 (0.12–0.14)
25–29	893,259	1927	0.55 (0.52–0.57)	935,255	706	0.19 (0.18–0.21)
30–34	929,095	2206	0.58 (0.56–0.61)	972,259	1272	0.32 (0.30–0.34)
35–39	971,995	2412	0.60 (0.58–0.62)	1,013,893	1929	0.46 (0.44–0.48)
40–44	1,003,078	2790	0.68 (0.65–0.70)	1,040,689	2652	0.62 (0.60–0.65)
45–49	1,022,809	3266	0.79 (0.77–0.82)	1,058,651	3618	0.85 (0.82–0.88)
50–54	1,002,258	3467	0.85 (0.83–0.88)	1,029,410	4652	1.12 (1.09–1.15)
55–59	925,235	3967	1.05 (1.01–1.08)	935,699	5703	1.50 (1.46–1.54)
60–64	849,920	5082	1.48 (1.44–1.52)	837,997	7029	2.09 (2.04–2.14)
65–69	767,425	6600	2.19 (2.13–2.24)	726,802	7610	2.69 (2.63–2.75)
70–74	671,501	7449	2.98 (2.91–3.05)	594,091	7425	3.44 (3.36–3.51)
75–79	544,360	8002	3.94 (3.86–4.03)	431,649	6416	4.16 (4.06–4.26)
80–84	430,778	7592	4.94 (4.83–5.05)	290,458	4772	4.92 (4.78–5.06)
85–89	296,681	5502	5.59 (5.44–5.74)	159,768	2684	5.58 (5.37–5.79)
90–94	153,877	2477	5.70 (5.47–5.92)	61,578	848	5.52 (5.14–5.89)
95–99	47,600	611	5.73 (5.27–6.18)	13,635	134	4.98 (4.13–5.82)
≥ 100	7179	44	3.65 (2.57–4.73)	1402	13	5.88 (2.68–9.07)
Total	12,864,571	65,401	1.28 (1.27–1.29)	12,560,372	58,142	1.17 (1.16–1.18)

### Death rates

**Fig. 2:**
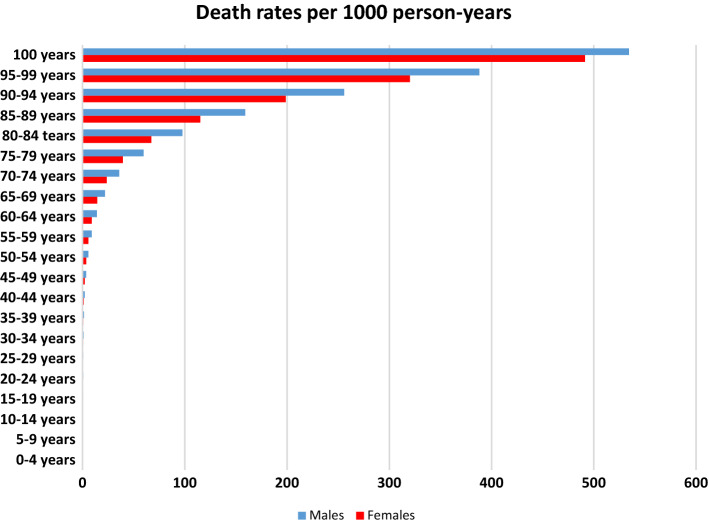
Death rates in men and women per 1000 person-years

Death rates in men and women by 5-year age groups are shown in Fig. 2. The death rates were essentially the same for men and women up to the age of 40 and then were consistently higher in men.

### Cumulative incidence of VTE

**Fig. 3:**
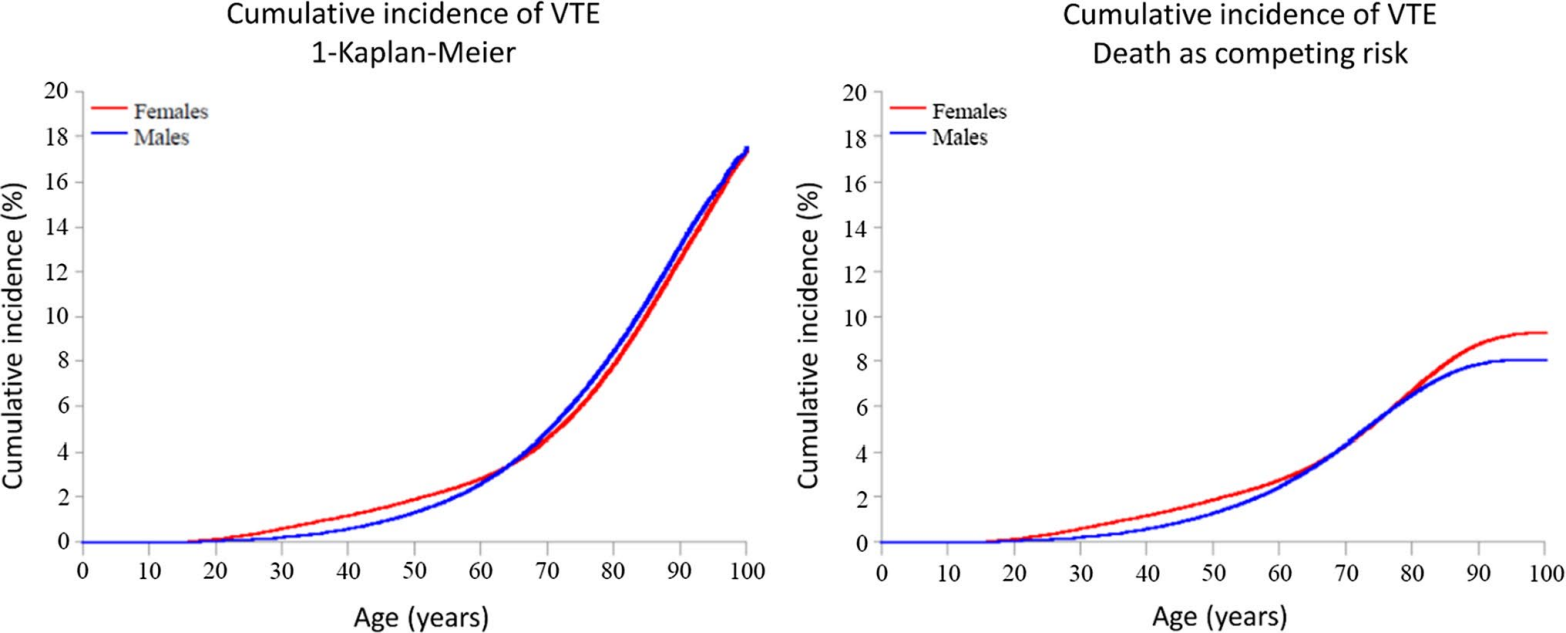
Cumulative incidence of venous thromboembolism (VTE) by increasing age in men and women, in analyses using traditional censoring (1-Kaplan Meier) (Panel A) and including death as a competing event (Panel B)

With the traditional 1-KM approach, the total cumulative incidences of VTE in men and women were 0.2% and 0.6% at age 30, 1.3% and 1.9% at age 50, 4.9% and 4.6% at age 70, and 17.6% and 17.4% at age 100, respectively (Fig. 3a). With the CIF approach, accounting for the competing risk of death, the estimated cumulative incidence of VTE was substantially lowered after the age of 65 in both sexes. The CIF estimates in men and women were 0.2% and 0.6% at age 30, 1.3% and 1.9% at age 50, 4.3% and 4.3% at age 70, and 8.1% and 9.3% at age 100, respectively (Fig. 3b).

**Fig. 4:**
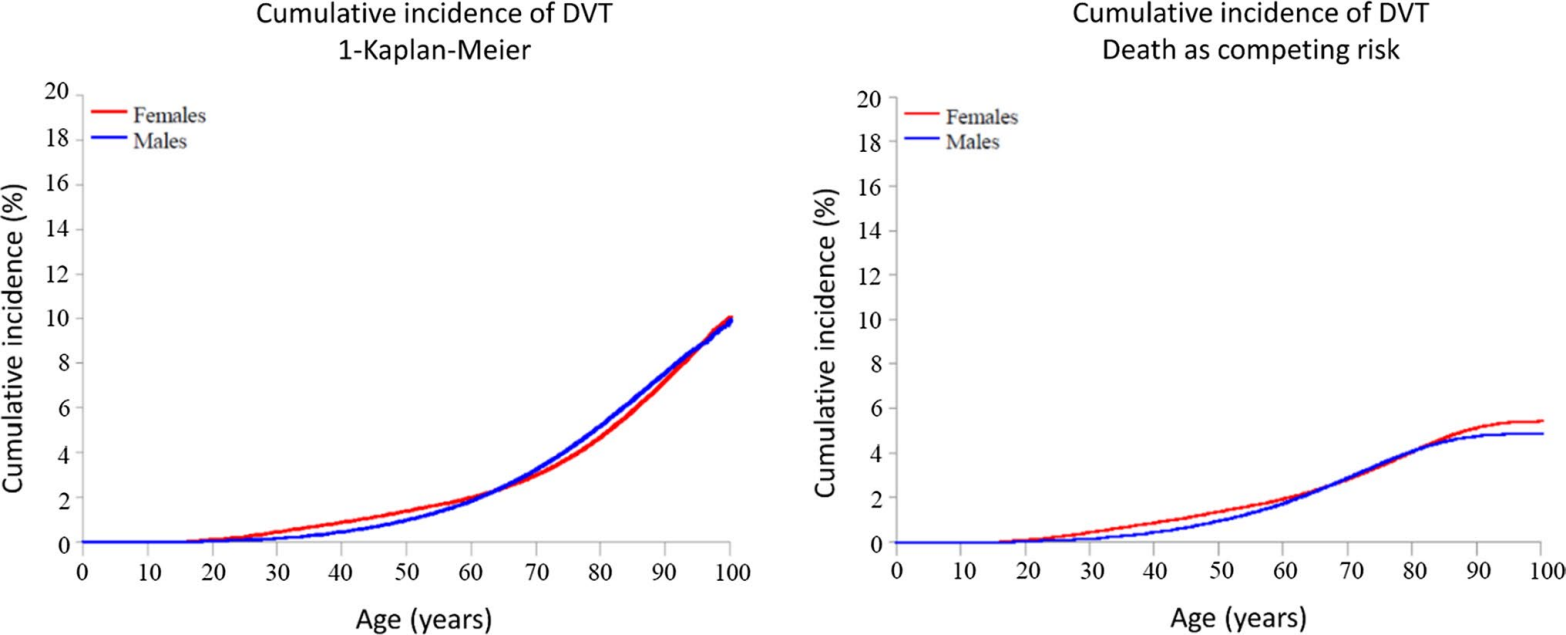
Cumulative incidence of deep vein thrombosis (DVT) by increasing age in men and women in analyses using traditional censoring (1-Kaplan Meier) (Panel A) and including death as a competing event (Panel B)

**Fig. 5:**
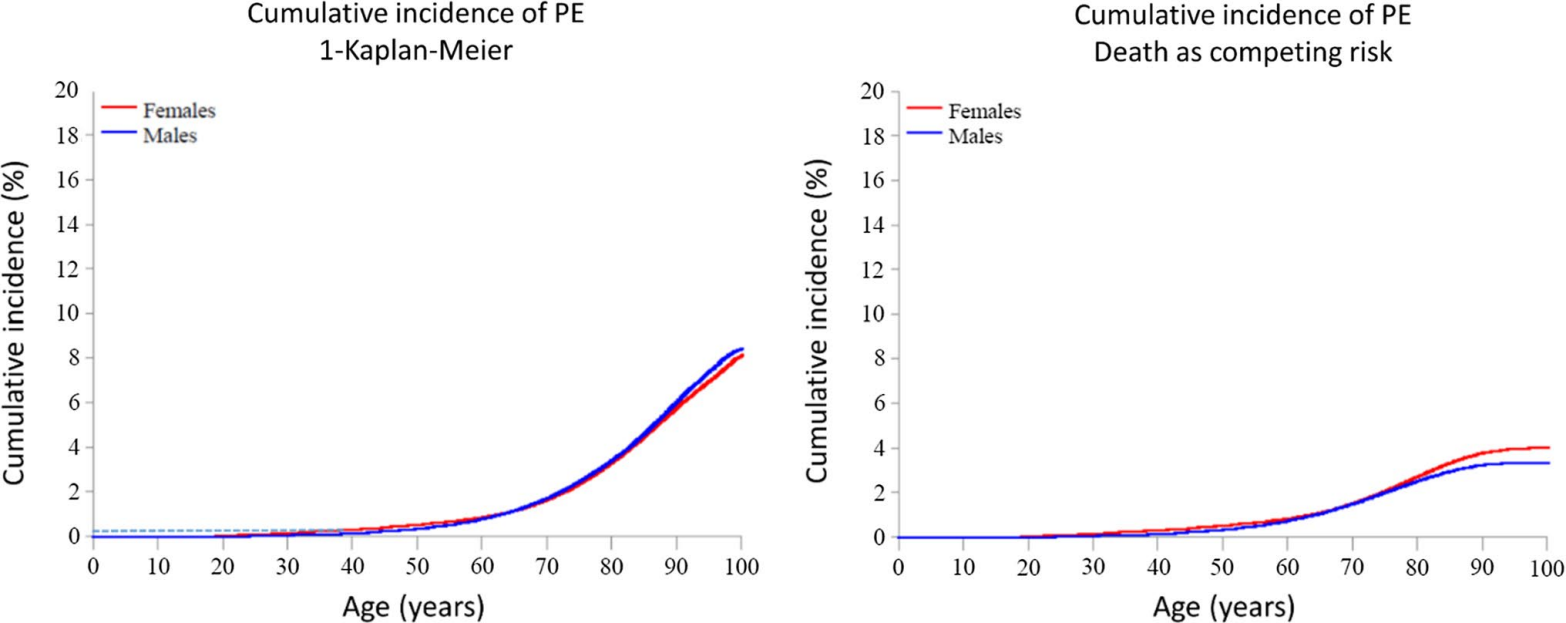
Cumulative incidence of pulmonary embolism (PE) by increasing age in men and women in analyses using traditional censoring (1-Kaplan Meier) (Panel A) and including death as a competing event (Panel B)

In subgroup analyses of DVT and PE, the 1-KM and CIF estimates followed the same patterns as those observed for overall VTE in men and women. With the 1-KM approach, the cumulative incidence of DVT at age 100 was similar in men (10.0%) and women (10.1%) (Fig. 4a). With the CIF approach the estimated cumulative incidence was marginally higher in women (5.4%) than in men (4.9%) (Fig. 4b). A similar pattern was observed for PE: the cumulative incidence at age 100 was 8.1% in women and 8.5% in men in the regular 1-KM analysis, and 4.0% in women and 3.3% in men in the CIF analysis (Fig. 5b).

Our sensitivity analysis showed that the CIF estimates of VTE were higher in both sexes during 2006–2016 compared to 1995–2005. Nevertheless, the patterns were similar in men and women in the two intervals, indicating no gender differences due to birth cohort effects (Supplementary Figs. 1a & b).

### Simulation study of cumulative incidence of VTE to account for female reproductive risk factors

**Fig. 6:**
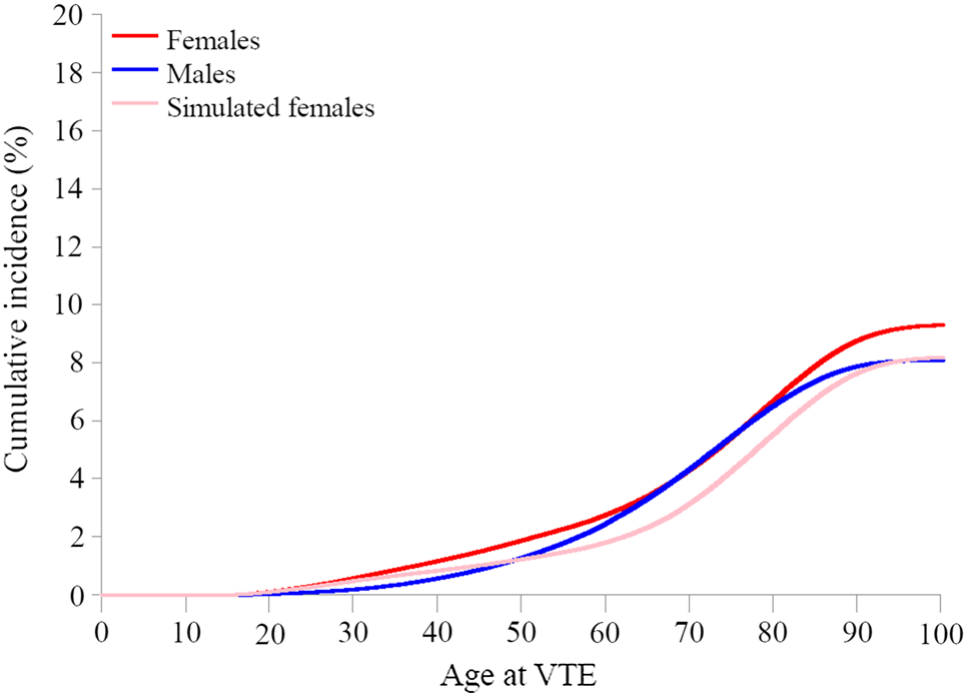
Cumulative incidence of venous thromboembolism (VTE) by increasing age in men and women including death as a competing risk, and simulated data on females excluding VTE cases caused by reproductive risk factors

In our simulation study, in which we ignored 45% of VTEs occurring in the female population aged 15–49 years (the proportion of events that could be attributed to female reproductive factors), the cumulative incidence of VTE was 0.5% at age 30, 1.2% at age 50, 3.2% at age 70, and 8.2% at age 100 (Fig. 6) after accounting for the competing risk of death.

## Discussion

We assessed age-specific cumulative incidence and estimated lifetime risk of VTE in men and women from the entire Danish population from 1995 to 2016, using an approach accounting for the competing risk of death. The estimated lifetime risk of VTE (cumulative incidence of VTE at age 100), was 9.3% in women and 8.1% in men based on incidence rates from a time period of approximately 20 years. Correspondingly, the estimated lifetime risk of DVT was 5.4% in women and 4.9% in men, while the estimated lifetime risk of PE was 4.0% in women and 3.4% in men. In the simulation study, in which we ignored the proportion of VTEs that could be attributed to reproductive risk factors in women, we found that the estimated lifetime risk was similar in men and women (8.1% and 8.2%, respectively). Our results indicate that the overall lifetime risk of VTE is marginally higher in women than in men after accounting for sex differences in death rates. The simulation study suggests that while female reproductive risk factors influence VTE risk in young women, they have a modest influence on their overall lifetime VTE risk.

Previous studies on sex-specific VTE risk have reported that men are either equally [5] or more at risk of VTE than women [24]. Since the incidence rates of VTE vary substantially across different age groups in men and women, the comparison of rates between the sexes is highly dependent on the age range of the population under study [18]. Moreover, when the rate of death is high from causes other than the disease of interest, the incidence rates of the disease are generally overestimated in traditional Kaplan–Meier survival analysis due to competing risks. To compare the lifetime risk of VTE in men and women, we therefore performed our analyses on an unselected nationwide cohort of women and men of all ages, considering death as a competing event. We found that the 1-KM estimate at age 100 was highly overestimated in both sexes. This is likely explained by the high death rate combined with the long follow-up in the study, as these parameters are known to increase the overestimation [12, 18]. As expected, the 1-KM estimate was more overestimated in men than in women due to the higher mortality rate in men.

We are aware of only one previous study that estimated cumulative VTE incidence while accounting for higher death rates in men than in women. Bell et al. estimated the lifetime risk of VTE in a cohort of 19,599 men and women aged 45–85 years with death as a competing risk [13]. In line with our findings, they reported a lifetime risk of VTE at age 85 of 8.4% in women and 7.7% in men [13], indicating that the overall lifetime incidence of VTE is marginally higher in women than in men, after accounting for death as a competing event.

The rapid increase in cumulative incidence observed in women in their early twenties may be explained by exposure to female reproductive risk factors, such as contraception and pregnancy. In men, we observed a steep increase in VTE incidence after age 50. At age 65 the proportion of men and women who had experienced a VTE was similar. A case–control study of persons aged 18–70 reported that the risk of a first VTE was twice as high in men than in women, after excluding females with reproductive risk factors [8]. However, the study included few females without reproductive risk factors, was based on relative measures, and the upper age limit was 70 years. Our study showed that half of all VTEs in the female population occurred after the age of 70, *i.e*., at an age when women are not exposed to reproductive risk factors. Moreover, only 22% of VTEs in women occurred before age 50. Based on previous literature [22], we estimated that approximately 45% of all VTEs in this age group could be attributed to oral contraceptives and pregnancy. In our simulation, in which we treated the proportion of cases among young women that could be attributed to reproductive risk factors as if they had not occurred, we found that the estimated lifetime risk was 8.2% in women. This was similar to the estimated lifetime risk in men of 8.1%. This indicates that even if VTEs occurring in women due to reproductive risk factors were avoided, the risk of VTE, taking a lifetime perspective, is similar in men and women.

A paper by Scheres et al. [25] based on the MEGA, Hokusai-VTE, and RIETE studies reported sex differences by the anatomical location of first VTE. A higher proportion of women than men presented with PE, with the difference ranging from 3.1 to 8.5 percentage points [25]. Of note, their findings were presented as conditional proportions, and not as absolute risks, which are needed to fully elucidate potential sex differences in presenting location. Like Scheres et al., we found a two-percentage-point higher proportion of PE among women (41.9%) than among men (39.1%) when considering all VTEs in the population. However, when expressed as absolute risks, the estimated lifetime risk was only marginally higher in women than in men for both PE (4.0% vs. 3.4%) and DVT (5.4% vs. 4.9%). Thus, our findings are in line with previous studies showing that the overall incidence of DVT and PE do not differ greatly in men and women [1, 2, 4, 6, 26].

The main strength of our study was use of an unselected nationwide cohort, which reduced the risks of selection and attrition bias that might occur in more traditional cohort studies. Moreover, VTE diagnoses in Denmark are usually made by trained specialists, and a validation study of DNPR data reported positive predictive values of 86% and 90% for diagnoses of first-time DVT and PE [27], further strengthening our findings. The study cohort consisted mostly of a homogenous Caucasian population, which reduced the likelihood of confounding by ethnicity, but also may limit its generalizability beyond this population. Another concern is that the slight increase in incidence of VTE during recent decades could be different in men and women [20]. However, a Danish study on temporal trends from 2006 to 2015 reported the same number of new VTE cases in men and women [20], and our sensitivity analyses showed that cumulative incidence patterns in men versus women were very similar in the first (1995–2005) and second (2006–2016) intervals of the study period. Unfortunately, we could not follow a closed cohort from birth to death, but estimated lifetime risk in a Nationwide cohort of all ages followed over 20 years. Even though we used age as time scale, included participants within the entire age range, and assessed whether there were sex-differences in cumulative incidence during follow-up, we cannot rule out potential presence of birth cohort effects that could differ in men and women. Another potential weakness of our study is that precise information on pregnancy and oral contraceptive use was not available in our database. However, Lidegaard et al.*’s* studies, which are derived from the same source population, indicated that 35–45% of VTEs in the 15–49 age group could be attributed to reproductive risk factors. Our simulation approach may be justified further by the exposure of the majority of young women either to oral contraceptive use and/or pregnancy during their lifetime. Restriction of the study population to women who were never exposed to such factors would result in a small and extremely selected population, which is unlikely to be representative of young women in general.

In conclusion, we found that when the competing risk of death was taken into account, the estimated lifetime risk of VTE, DVT and PE was marginally higher in women than in men. Furthermore, when VTEs that could be attributed to female reproductive risk factors were accounted for in a simulation study, the estimated lifetime risk of VTE was similar in men and women, indicating that the contribution of reproductive risk factors to the overall lifetime risk of VTE in women is modest. Our findings challenge previous studies reporting a twofold higher risk of first-time VTE in men compared to women without reproductive risk factors, and studies reporting a higher proportion of PEs in women than in men. Finally, our observation of 8–10% estimated lifetime risk of VTE in women and men highlights the importance of improved primary prevention in both sexes.

## Supplementary Information

Below is the link to the electronic supplementary material.

Supplementary file 1 (DOCX 185 KB)

## Data Availability

All data were obtained from Danish healthcare and administrative registries.

## Code availability

SAS version 9.4 (SAS Institute Inc. Cary, North Carolina, USA).
