# Supplementary material for: Estimated lifetime risk of venous thromboembolism in men and women in a Danish nationwide cohort: impact of competing risk of death
Source: Eur J Epidemiol. 2021 Nov 8;37(2):195–203. doi: 10.1007/s10654-021-00813-w (PMC8960584; doi:10.1007/s10654-021-00813-w)
Supplement: Supplementary file 1 — (DOCX 185 KB) [file 10654_2021_813_MOESM1_ESM.docx]

**Supplementary Figure 1.** Cumulative incidence of venous thromboembolism (VTE), among cases identified during the entire follow up-period compared to cases identified during 1995-2005 (Panel 1A) and cases identified during 2006-2016 (Panel 1B).


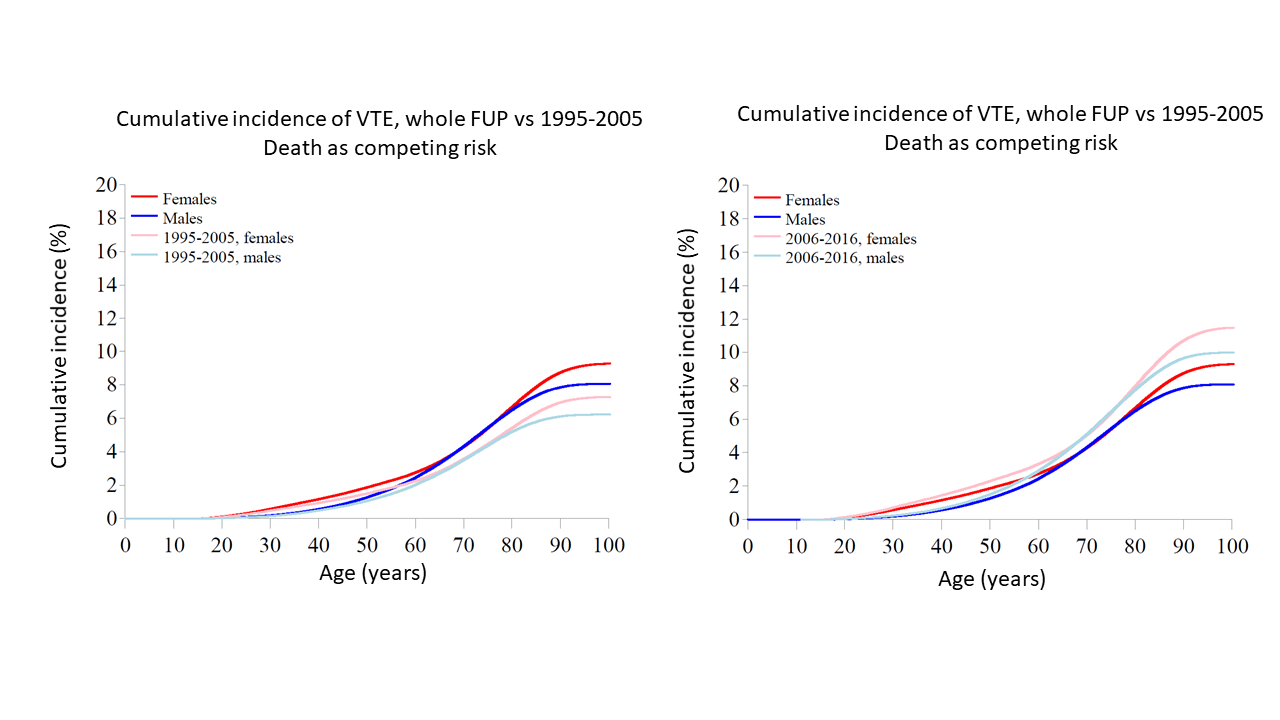


**Supplementary Table 1.** Male and female DVT outcomes and Incidence rates by five-year age groups.

|  | **Women** | | | **Men** | | |
| --- | --- | --- | --- | --- | --- | --- |
| **Age (years)** | **n** | **Events** | **IR (95% CI)** | **n** | **Events** | **IR (95% CI)** |
| 0-4 | 167,754 | 2 | 0.00 (-0.00–0.01) | 176,096 | 0 | - |
| 5-9 | 313,930 | 5 | 0.00 (0.00–0.01) | 329,681 | 6 | 0.00 (0.00–0.01) |
| 10-14 | 451,067 | 21 | 0.01 (0.01–0.02) | 472,786 | 15 | 0.01 (0.00–0.01) |
| 15-19 | 614,941 | 467 | 0.17 (0.16–0.19) | 643,523 | 155 | 0.06 (0.05–0.06) |
| 20-24 | 799,829 | 981 | 0.27 (0.25–0.29) | 835,050 | 346 | 0.10 (0.09–0.11) |
| 25-29 | 893,259 | 1,462 | 0.36 (0.34–0.38) | 935,255 | 534 | 0.15 (0.13–0.16) |
| 30-34 | 929,095 | 1,628 | 0.37 (0.35–0.39) | 972,259 | 987 | 0.25 (0.23–0.26) |
| 35-39 | 971,995 | 1,752 | 0.41 (0.39–0.43) | 1,013,893 | 1,454 | 0.35 (0.33–0.37) |
| 40-44 | 1,003,078 | 1,952 | 0.47 (0.45–0.49) | 1,040,689 | 1,914 | 0.45 (0.43–0.47) |
| 45-49 | 1,022,809 | 2,285 | 0.56 (0.53–0.58) | 1,058,651 | 2,599 | 0.61 (0.59–0.64) |
| 50-54 | 1,002,258 | 2,348 | 0.58 (0.56–0.60) | 1,029,410 | 3,231 | 0.78 (0.75–0.81) |
| 55-59 | 925,235 | 2,498 | 0.66 (0.63–0.68) | 935,699 | 3,693 | 0.97 (0.94–1.00) |
| 60-64 | 849,920 | 3,042 | 0.88 (0.85–0.92) | 837,997 | 4,427 | 1.32 (1.28–1.35) |
| 65-69 | 767,425 | 3,580 | 1.19 (1.15–1.22) | 726,802 | 4,449 | 1.57 (1.53–1.62) |
| 70-74 | 671,501 | 3,868 | 1.55 (1.50–1.59) | 594,091 | 4,081 | 1.89 (1.83–1.95) |
| 75-79 | 544,360 | 4,038 | 1.99 (1.93–2.05) | 431,649 | 3,411 | 2.21 (2.14–2.28) |
| 80-84 | 430,778 | 3,836 | 2.49 (2.42–2.57) | 290,458 | 2,413 | 2.49 (2.39–2.59) |
| 85-89 | 296,681 | 2,814 | 2.86 (2.75–2.96) | 159,768 | 1,250 | 2.60 (2.45–2.74) |
| 90-94 | 153,877 | 1,377 | 3.17 (3.00–3.33) | 61,578 | 392 | 2.55 (2.30–2.80) |
| 95-99 | 47,600 | 336 | 3.15 (2.81–3.49) | 13,635 | 65 | 2.41 (1.83–3.00) |
| ≥100 | 7,179 | 28 | 2.32 (1.46–3.19) | 1,402 | 7 | 3.16 (0.82–5.51) |
| Total | 12,864,571 | 38,320 | 0.75 (0.74-0.76) | 12,560,372 | 35,429 | 0.71 (0.70-0.72) |

**Supplementary Table 2.** Male and female PE outcomes and incidence rates by five-year age groups.

|  | **Women** | | | | **Men** | | |
| --- | --- | --- | --- | --- | --- | --- | --- |
| **Age (years)** | **n** | **Events** | | **IR (95% CI)** | **n** | **Events** | **IR (95% CI)** |
| 0-4 | 167,754 | 0 | | - | 176,096 | 0 | - |
| 5-9 | 313,930 | 0 | | - | 329,681 | 1 | 0.00 (-0.00–0.00) |
| 10-14 | 451,067 | 4 | | 0.00 (0.00–0.00) | 472,786 | 2 | 0.00 (-0.00–0.00) |
| 15-19 | 614,941 | 153 | | 0.06 (0.05–0.07) | 643,523 | 39 | 0.01 (0.01–0.02) |
| 20-24 | 799,829 | 374 | | 0.11 (0.10–0.12) | 835,050 | 115 | 0.03 (0.03–0.04) |
| 25-29 | 893,259 | 465 | | 0.13 (0.12–0.14) | 935,255 | 172 | 0.05 (0.04–0.05) |
| 30-34 | 929,095 | 578 | | 0.15 (0.14–0.16) | 972,259 | 285 | 0.07 (0.06–0.08) |
| 35-39 | 971,995 | 660 | | 0.16 (0.15–0.18) | 1,013,893 | 475 | 0.11 (0.10–0.12) |
| 40-44 | 1,003,078 | 838 | | 0.20 (0.19–0.22) | 1,040,689 | 738 | 0.17 (0.16–0.19) |
| 45-49 | 1,022,809 | 981 | | 0.24 (0.22–0.25) | 1,058,651 | 1,019 | 0.24 (0.23–0.25) |
| 50-54 | 1,002,258 | 1,119 | | 0.28 (0.26–0.29) | 1,029,410 | 1,421 | 0.34 (0.33–0.36) |
| 55-59 | 925,235 | 1,469 | | 0.39 (0.37–0.41) | 935,699 | 2,010 | 0.53 (0.50–0.55) |
| 60-64 | 849,920 | 2,040 | | 0.59 (0.57–0.62) | 837,997 | 2,602 | 0.77 (0.74–0.80) |
| 65-69 | 767,425 | 3,020 | | 1.00 (0.96–1.04) | 726,802 | 3,161 | 1.12 (1.08–1.16) |
| 70-74 | 671,501 | 3,581 | | 1.43 (1.38–1.48) | 594,091 | 3,344 | 1.55 (1.49–1.60) |
| 75-79 | 544,360 | 3,964 | | 1.95 (1.89–2.02) | 431,649 | 3,005 | 1.95 (1.88–2.02) |
| 80-84 | 430,778 | 3,756 | | 2.44 (2.37–2.52) | 290,458 | 2,359 | 2.43 (2.33–2.53) |
| 85-89 | 296,681 | 2,688 | | 2.73 (2.63–2.83) | 159,768 | 1,434 | 2.98 (2.83–3.13) |
| 90-94 | 153,877 | 1,100 | | 2.53 (2.38–2.68) | 61,578 | 456 | 2.97 (2.69–3.24) |
| 95-99 | 47,600 | 275 | | 2.58 (2.27–2.88) | 13,635 | 69 | 2.56 (1.96–3.17) |
| ≥100 | 7,179 | 16 | | 1.33 (0.68–1.98) | 1,402 | 6 | 2.71 (0.54–4.88) |
| Total | 12,864,571 | 27,081 | 0.53 (0.52-0.54) | | 12,560,372 | 22,713 | 0.46 (0.45-0.46) |
